# PymoNNto: A Flexible Modular Toolbox for Designing Brain-Inspired Neural Networks

**DOI:** 10.3389/fninf.2021.715131

**Published:** 2021-11-01

**Authors:** Marius Vieth, Tristan M. Stöber, Jochen Triesch

**Affiliations:** Frankfurt Institute for Advanced Studies, Frankfurt am Main, Germany

**Keywords:** neural network simulator, software toolbox, python library, graphical user interface (GUI), simulator fusion, evolutionary algorithm

## Abstract

The Python Modular Neural Network Toolbox (PymoNNto) provides a versatile and adaptable Python-based framework to develop and investigate brain-inspired neural networks. In contrast to other commonly used simulators such as Brian2 and NEST, PymoNNto imposes only minimal restrictions for implementation and execution. The basic structure of PymoNNto consists of one network class with several neuron- and synapse-groups. The behaviour of each group can be flexibly defined by exchangeable modules. The implementation of these modules is up to the user and only limited by Python itself. Behaviours can be implemented in Python, Numpy, Tensorflow, and other libraries to perform computations on CPUs and GPUs. PymoNNto comes with convenient high level behaviour modules, allowing differential equation-based implementations similar to Brian2, and an adaptable modular Graphical User Interface for real-time observation and modification of the simulated network and its parameters.

## 1. Introduction

Simulating neural networks has become an indispensable part of brain research, allowing neuroscientists to efficiently develop, explore, and evaluate hypotheses. Working with such models is facilitated by various simulation environments, which typically provide high level classes and functions for convenient model generation, simulation, and analysis.

Each simulation environment has particular strengths and limitations. Neural network models can be formulated at different levels of detail/abstraction. Reflecting the various scales of investigation, several simulation environments exist, each with its own focus area (for review see Brette et al., 2007; Brette and Goodman, 2012; Tikidji-Hamburyan et al., 2017). While for example, *Neuron* (Hines and Carnevale, 1997) excels at simulating neurons with a high degree of biological detail, *NEST* (Fardet et al., 2020) is optimized to simulate large networks of rather simplified spiking neurons on distributed computing clusters (Jordan et al., 2018). Another simulator, *Brian*/*Brian2* (Goodman and Brette, 2009; Stimberg et al., 2019) prioritizes concise model definition over scaling to large computing environments.

Typically, the convenience provided by a particular neural network simulation toolbox comes at the price of reduced flexibility. This can cause problems when researchers need to leave the “comfort zone” of a particular simulator. For example, when aiming to explore a novel plasticity rule, investigators may be confronted with a difficult choice: They either have to work their way around the constraints of the simulator or write their own simulation environment from scratch. While implementing a workaround may turn out to be arduous and complicated, writing a simulation environment from scratch is time consuming, error prone, hampering reproducibility, and sacrificing useful features of mature simulation environments (Pauli et al., 2018).

The scientific community has become increasingly aware of this dilemma. Several developments aim to increase the flexibility of existing simulators. For example, NEST has been extended with its own modeling language to allow for custom model definition without having to write C++ modules (Plotnikov et al., 2016). Brian2 simulations, limited to a single core, can be accelerated by executing them on GPUs (Stimberg et al., 2020) via automated code translation to GeNN (Yavuz et al., 2016). However, in all cases, specific simulator-inherent restrictions remain.

An alternative strategy to achieve both flexibility and reproducibility is to detach model definition from its execution. Simulator-independent model description interfaces, such as PyNN (Davison et al., 2009) or general model description languages, such as NeuroML (Gleeson et al., 2010), allow to first specify a model using a fixed set of vocabulary and syntax. In a second step, model definition is automatically translated to a selected simulation environment. In either approach flexibility remains bounded: The ability to express new mechanisms is limited by a finite number of language elements and the restrictions of the available simulation environments.

**Figure 1:**
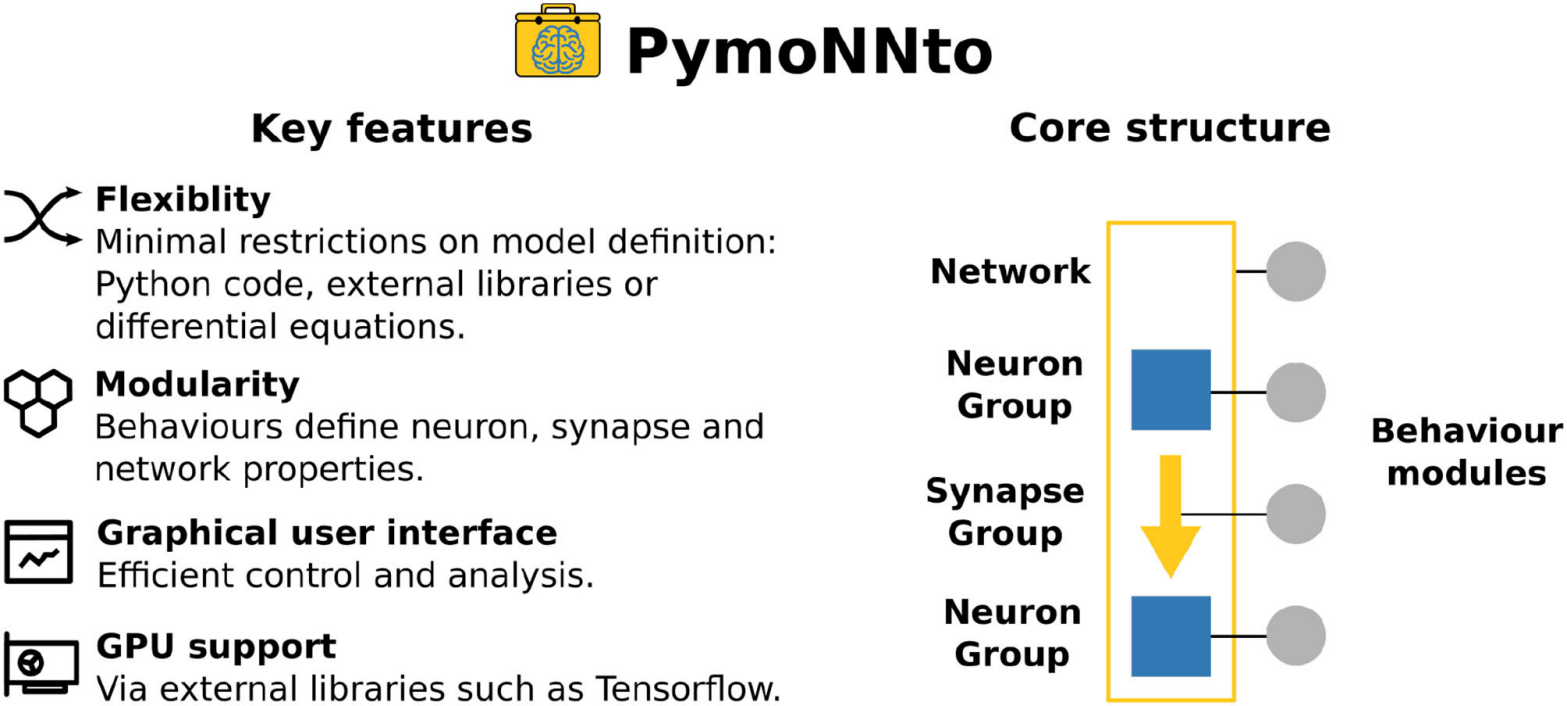
Key features of PymoNNto **(left)** and its core structure **(right)**. The core structure consists of a *Network* class, *NeuronGroups, SynapseGroups*, and *Behaviour* modules. One *Network* can have many *NeuronGroups* and *SynapseGroups*. One or more *Behaviour* modules with custom code are attached to all three classes.

To address the dilemma between flexibility and convenience with a novel approach, we designed PymoNNto as a modular low level Python (Van Rossum and Drake, 1995) framework with minimal restrictions, while at the same time providing several high level modules for convenient analysis and interaction (see Figure 1 for an overview of PymoNNto's key features and core structure). Its lightweight structure comes with a number of advantages: (1) Dynamics of neurons and synapses can be freely designed by custom behaviour modules. (2) The content of behaviour modules is only limited by the expressive power of Python. (3) These modules can be optimized for speed, for example via Tensorflow (Abadi et al., 2016) or Cython (Behnel et al., 2010), and can even wrap around and combine established simulators, facilitating multi-scale approaches. Without sacrificing flexibility, PymoNNto allows for efficient implementation and analysis via a multitude of features, such as a powerful and extendable graphical user interface, a storage manager, and several pre-implemented neuronal/synaptic mechanisms and network models (compare Table 1).

**Table 1:** Pre-implemented neuronal mechanisms and network models.

**Neuronal/synaptic mechanisms**
Spike-timing-dependent plasticity (STDP) (Lazar et al., 2009)
Synaptic weight normalization (Lazar et al., 2009)
Intrinsic plasticity (IP) (modified from Lazar et al., 2009)
Refractory period
NOX diffusion-based homeostasis (Sweeney et al., 2015)
**Network/Neuron Models**
Hodgkin and Huxley (1952)
Hopfield (1982)
Hindmarsh and Rose (1984)
Wang and Buzsáki (1996)
Brunel and Hakim (1999)
Diesmann et al. (1999)
Izhikevich (2003)
Brunel and Hakim (1999)

## 2. Architecture and Functionality

To streamline the network development workflow, the core of PymoNNto forms a scaffold in which the user can embed his own code. In short, this scaffold consists of a network containing neurons and synapses. Interactions between these elements are defined by behaviour modules. The main purpose of this scaffold is to add structure to the model, to simplify the development process through communication functions and to make the development of additional tools more convenient.

PymoNNto's architecture aims to represent neural circuits by reusable building blocks in an object-oriented fashion. The dynamics of each building block are described by a behaviour module—representing for example a specific synaptic receptor class. PymoNNto's modular design allows for efficient addition or removal of such building blocks, and thus facilitates the development and investigation of complex neural networks.

### 2.1. Core Classes

**Figure 2:**
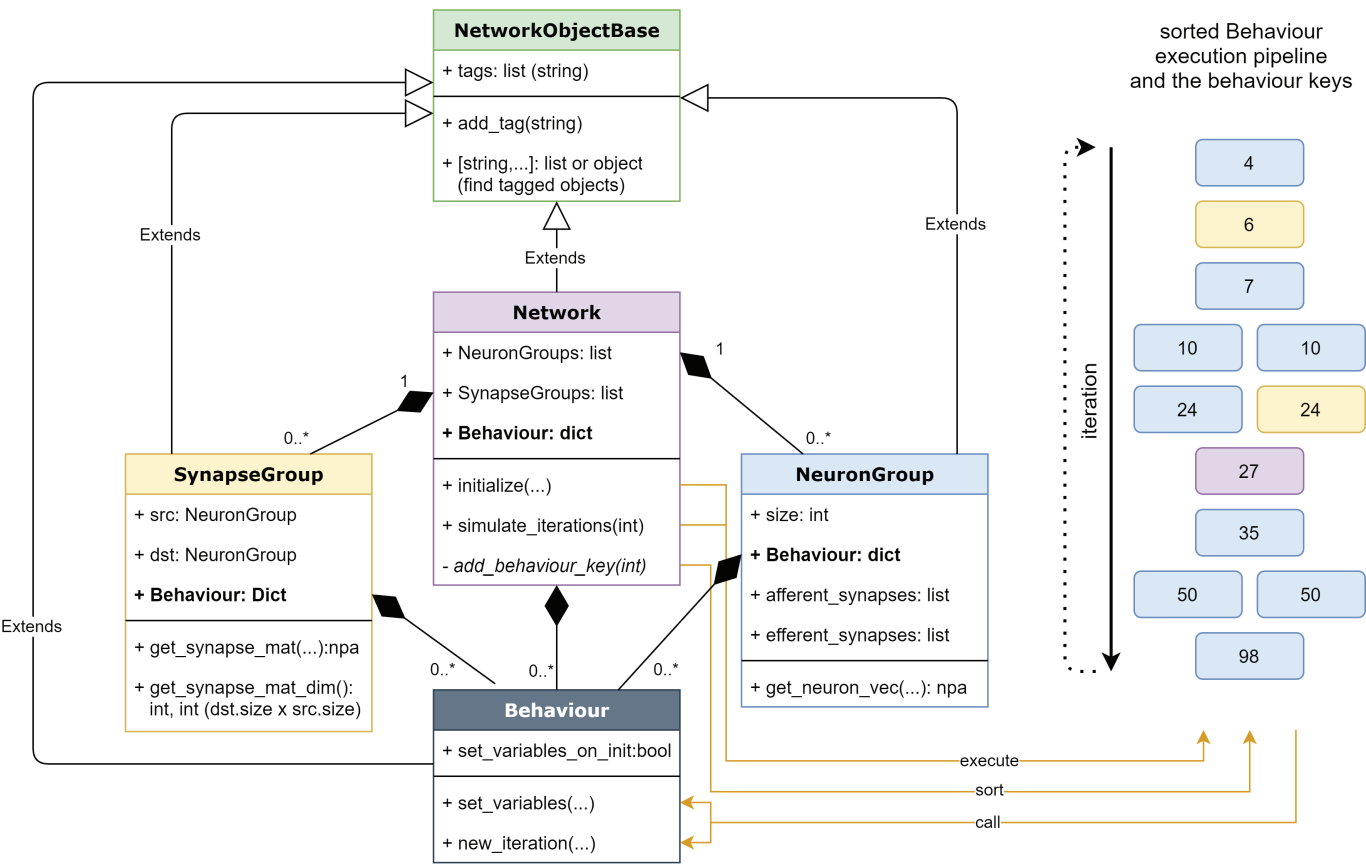
A UML Diagram of PymoNNto's core with its most relevant variables and functions on the left as well as a visualization of an example execution pipeline composed of sorted behaviours (boxes) on the right. The white arrows indicate inheritance relationships and the black diamonds indicate compositions. The numbers in the execution pipeline represent the keys of corresponding behaviours in the dictionary of their parent object. The colors of these behaviours correspond to the colors of their parents on the left. The orange arrows connect both visualizations and indicate how the functions interact with the execution pipeline. npa, NumPy array.

The low level core of PymoNNto consists of four main classes derived from the same *NetworkObjectBase* class. Figure 2 shows a detailed UML diagram explaining the inheritance relationships among the different classes. It also shows an example execution pipeline, where the behaviours have been sorted by their “keys” specifying the order of execution.

**NeuronGroup**

*NeuronGroup* objects represent populations of neurons. PymoNNto neurons have no neuron-like behaviour, logic, or data by default. They can be seen as empty shells, which can be filled with custom code modules. A *NeuronGroup* object contains a list of behaviour modules which define what the neurons are doing, what variables they have, and how they communicate with other *NeuronGroups*. Further, *NeuronGroup* objects are equipped with functions to efficiently access afferent and efferent synapses, to initialize vectors for data storage, and to partition the group into subgroups.

**SynapseGroup**

*SynapseGroups* are used to connect source and target *NeuronGroups*. As in *NeuronGroups, SynapseGroups* can be freely defined by their own behaviour modules.
In contrast to *NeuronGroups*, which contain functions to initialize activity vectors, *SynapseGroups* contain helper functions to initialize synaptic weight matrices with specific connection densities and receptive fields.

**Network**

The *Network* object is the main object and contains all *Neuron-* and *SynapseGroups* of the simulation, as well as some optional global behaviour modules. It provides mechanisms for communication between the groups, functions to control the simulation and manages the order of execution of the custom code blocks.

**Behaviour**

*Behaviour* modules are the core of the simulation and contain custom code. A *Behaviour* module is divided into an initialization- and an update-function called at every time step. Module-specific variables and functions can be stored inside, while shared functionality should be stored in the parent object.
The *Behaviour* modules can be initialized in a very compact way with different helper functions to define their attributes. This allows to describe the full network and associated parameters in one file. Behaviour modules can be associated to the *Network* object, *NeuronGroups*, or *SynapseGroups*. However, in most cases, the *NeuronGroup* objects are the preferred objects to which *Behaviour* modules are assigned. This facilitates operations across different *SynapseGroups*, such as a synaptic normalization mechanism that scales the sum of all excitatory synapses onto a neuron to a specific value. Because *Behaviour* modules are classes, they can benefit from all the advantages of object oriented programming, such as inheritance.

### 2.2. Internal Processing

The internal workings of PymoNNto's core are simple. When *Behaviour* modules are assigned to different objects (compare Code block 2), each of these modules receives an individual number which determines the order of execution. These behaviour numbers are sorted across all objects of the network during initialization. The main loop repeatedly executes all the behaviours in the determined order (see Figure 2), which only needs one dictionary access per behaviour.

### 2.3. Additional High Level Functions

In addition to the four core objects, PymoNNto contains a multitude of optional high level helper functions and tools to streamline network design and investigation. Here, we briefly summarize the most useful ones (see online documentation for more details):

**Graphical User Interface (GUI)**

**Figure 3:**
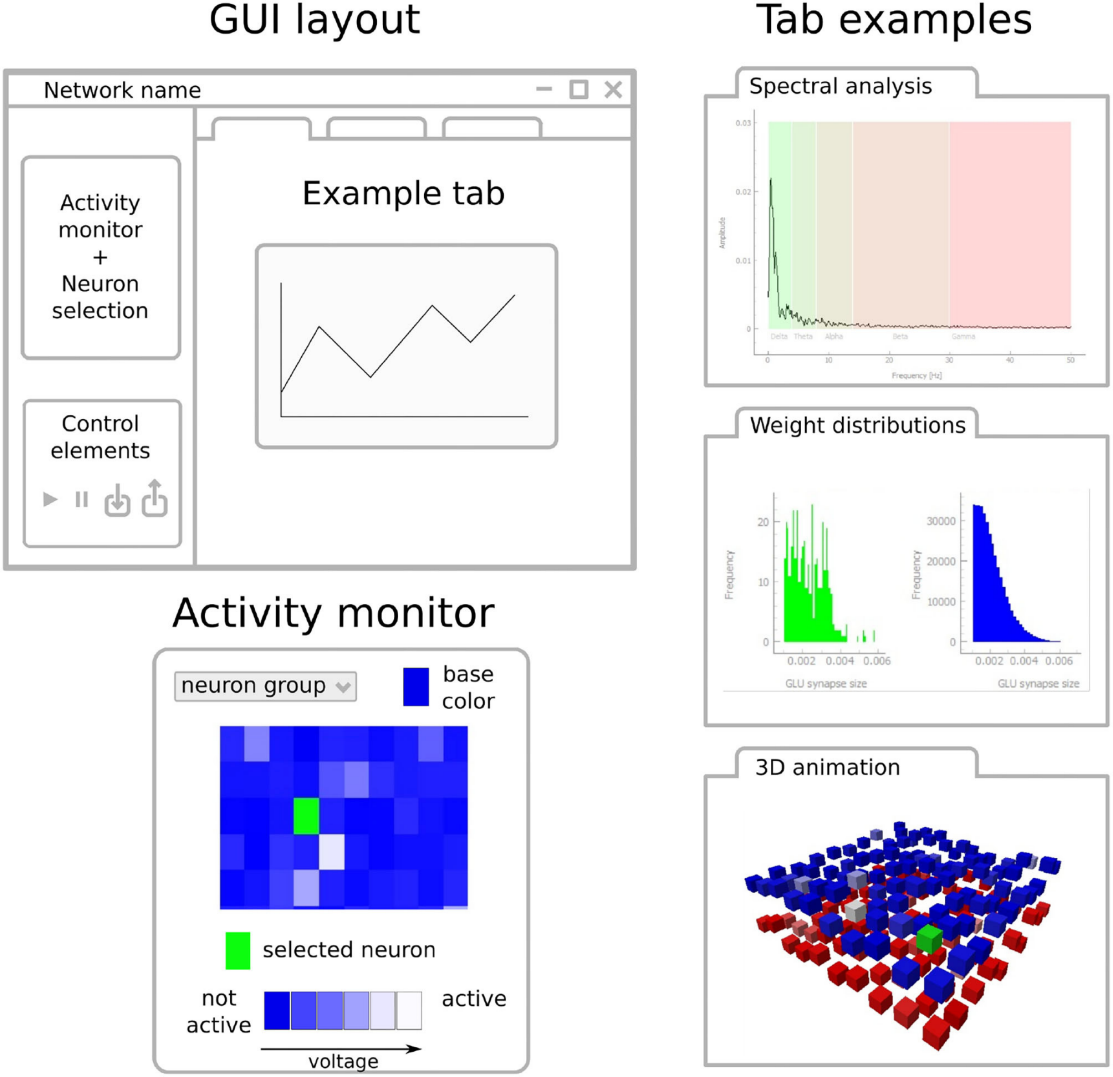
Design elements of PymoNNto's graphical user interface. The GUI layout is structured as follows: The left bar contains an activity monitor and control elements. The activity monitor displays one or several neuron groups in real time. Each neuron group can receive a distinctive base color. In addition, one can select one or several overlay colors to display ongoing activity, reflecting for example the current voltage or spikes. The activity monitor allows to select individual neurons (green) for further analysis. Control elements allow for example to start, pause, save, and load the simulation and can contain additional tab-specific elements. PymoNNto's GUI contains a large variety of tabs which can be used to analyze and monitor network properties. Three exemplary tabs are shown on the right: Spectral analysis of membrane potentials **(top)**, histograms of synaptic weight distributions **(center)**, and a three-dimensional animation of network activity with excitatory neurons in blue, inhibitory neurons in red **(bottom)**, recently active neurons in white, and a selected neuron in green. For a real GUI example, see **Figure 6**.

PymoNNto's GUI is a powerful tool to interactively explore the behaviour of a network simulation. Parameters can be modified and statistics displayed in real time. For example, as parameters are varied or plasticity mechanisms switched on or off, the GUI allows to monitor ongoing network activity, the presence of activity oscillations, or emerging changes to the network connectivity (see Figure 3). The GUI is organized into modular and customizable tabs. It is based on PyQt5 (Riverbank Computing, 2020) and uses additional PyQtGraph (Campagnola, 2020) elements for plotting.

**Tagging system**

To simultaneously access similar variables in multiple objects, the *NetworkObjectBase* class contains a tagging system. It can be used to find objects with the same tag, such as all *SynapseGroups* tagged with *Glutamate* receptors. The tagging systems helps to write simple, compact code by giving the programmer easy access to all tagged objects within an instance of a class. To use the tagging system the *MyObject[“tag”]* operator can be used. This removes the need to create variables for all kinds of objects and pass them to functions via multiple arguments. The only object that has to be passed is the root object, typically the network, and everything else can be accessed via the respective tag.

**Recorder**

The *Recorder* module records some custom variable of a *NeuronGoup* at a given interval. This allows PymoNNto to store activity traces for plotting and further analysis. The *Recorder* can not only record variables, but also results of custom functions. One can, for example, use the string “*n.activity”* to record the neurons' activity, but it is also possible to use “*f(n.activity,…)”*, where f can be the mean or the sum of the activity vector, for example. This is possible, because the string is compiled into code at runtime. Another useful feature is that this recording string can also be used as a tag for the previously described tagging system. For example, after adding a recorder with “*n.activity,”* calling *MyNetwork[“n.activity”]* will return a list of all recorded activity traces.

**Storage manager**

To store recording data, parameters, results and variables, a *Storage-Manager* is included in PymoNNto. It searches for a “Data” folder in the project directory and can create a directory with a custom name for a group of simulation runs. At every run, it creates a separate sub-folder to save and load vectors, matrices, images, videos, and parameters. Furthermore, the *Storage-Manager* allows to sort, compare, and analyse multiple runs with respect to different parameters of interest.

**Partitioning**

The partitioning function is helpful when designing locally-connected networks. When the implemented model is based on vector and matrix operations, the *NeuronGroups* can be divided into *SubNeuronGroups* with a mask. Such a *SubNeuronGroup* allows partial access to variables of the original *NeuronGroup*. The use of *SubNeuronGroups* can avoid slow computations due to large connection matrices by splitting one big sparse *SynapseGroup* into many smaller and denser ones.
When adding the partitioning behaviour module to a *SynapseGroup* it will automatically detect the pre- and the post-synaptic *NeuronGroup* as well as the maximal distance in which a neuron can make connections. This information is then used to replace the big *SynapseGroup* with multiple smaller ones that are attached to *SubNeuronGroups*. With this, we can conveniently combine fast processing with small computational overhead and avoid the quadratic growth of synaptic weight matrices for increasing numbers of neurons when using networks of locally connected neurons.

**Evolution**

PymoNNto's *Evolution* package follows generic evolutionary principles to optimize parameters (Eiben et al., 2003; Vikhar, 2016): Multiple networks are initiated as *individuals*, differing in selected parameters, so called *genes*. In each round of simulation, the fitness of each individual is evaluated by a scoring function, a fraction of individuals with the best score survive and new individuals are generated with mutated parent genes. To use the *Evolution* package, the user may insert the two functions get_gene(key, default) and set_score(score) as interfaces to receive new parameters and to set the fitness in a given simulation file. During the optimization process, this file is executed multiple times, either on different cores or machines (accessed via ssh). This process can be controlled either by a master file or by the *Evolution* package's own graphical user interface. For more details and a code example, we refer the reader to the PymoNNto's online documentation. Note, the general design of the *Evolution* package allow its use beyond the context of network simulations.

## 3. How To Use PymoNNto?

PymoNNto is based on Python3 and can be installed with the pip package installer with the command: “*pip install pymonnto”* We also refer the reader to the online documentation and the GitHub repository for more detailed examples and descriptions.

**Figure 4:**
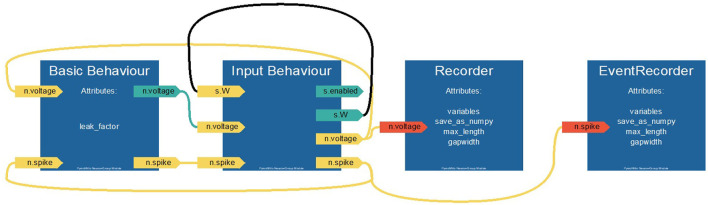
Automatically generated flow chart of the PymoNNto model defined in Code blocks 1–3. Interactions between *Basic_Behaviour* and *Input_Behaviour* as well as the respective recorders are shown. Positions of behaviour modules reflect the order of internal execution from left to right.

In the following, we demonstrate how to implement a minimal network with PymoNNto. The network consists of a group of simplified leaky-integrate and fire (LIF) neurons, communicating via excitatory synapses. To keep things simple, the resting and reset voltages of the simplified LIF neurons are defined to be zero. Membrane potential updates are calculated by numerically solving the differential equations with the Euler method for a fixed number of iterations. All code blocks in the section are compatible with each other. The relations between modules defined in Code blocks 1–3 are visualized in a flowchart, automatically generated via the function *My_Neurons.visualize_module()* (see Figure 4).

### 3.1. Basic Structure

The core of a PymoNNto simulation consists of three steps: (a) defining network, neurons, and synapses, (b) initializing, and (c) simulating them (see Code block 1). Both the *NeuronGroup* and the *SynapseGroup* receive as input the parent network and a name tag. Further, the *NeuronGroup* requires a size argument and the *SynapseGroup* its source and destination.


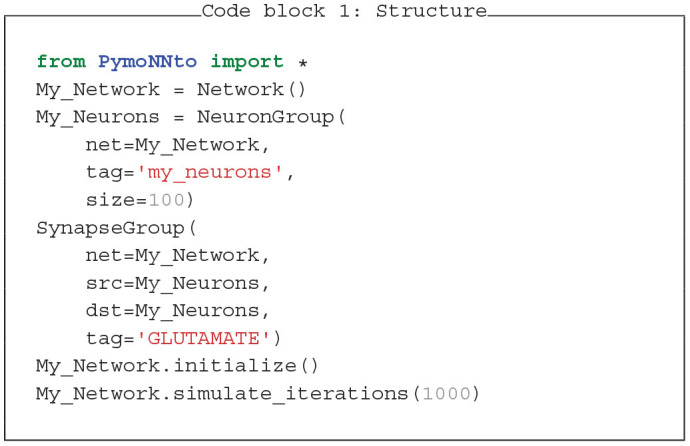


### 3.2. Behaviour

*Behaviour* modules allow to define custom dynamics of neurons and synapses. Each *Behaviour* module typically consists of two functions: *set_variables* is called when the Network is initialized and *new_iteration* is called every time step. Both functions receive an additional attribute, in code block 2 it is named *neurons*, which points to the group the behaviour belongs to, in this case a *NeuronGroup*. This attribute allows to use parent group specific functions and to define and modify its variables. In this example, we initialize the *NeuronGroup* variable *voltage* with zero values via the *get_neuron_vec* function. At every timestep, we add random membrane noise to these voltages with *get_neuron_vec(“uniform,”…)*. Further, we define a local variable *threshold*, defining the voltage above which the neuron will create a spike before being reset, as well as the variable *leak_factor* for the *voltage* reduction at each iteration. Here, it is not relevant whether variables are stored in the neuron- or the behaviour-object. Though, in more complex simulations it can be advantageous to store variables only used by the behaviour in the *Behaviour* object and other variables in the parent object.


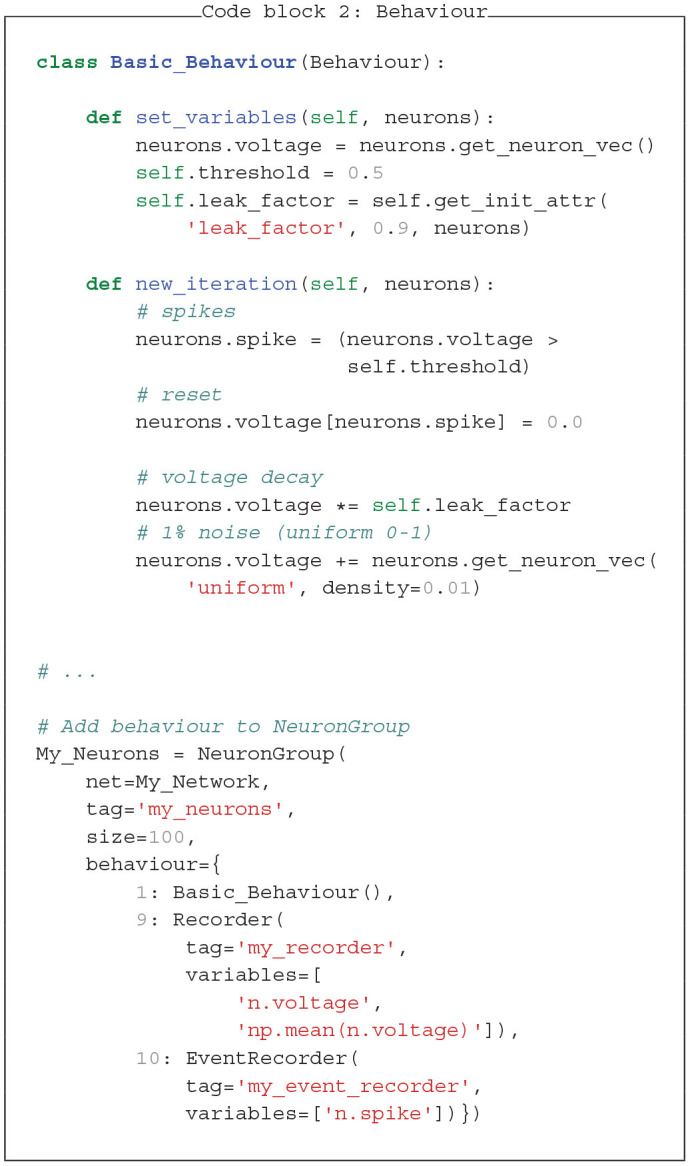


We add the *Basic_Behaviour* to the *NeuronGroup* together with a pre-defined *Recorder* behaviour, to store the *voltage* variable over time. Behaviours are added to a *NeuronGroup* or *SynapseGroup* as a dictionary. The key in front of each *behaviour* has to be a positive number and determines the order of execution across the whole simulation. Note, that correct ordering is important. At the initialization step all behaviours across all neuron or synapse groups are ordered. In case behaviours have the same index, the order is randomly set (see Figure 2). Here, we chose a higher number for the recorder, to store values at the end of each iteration.

### 3.3. Synapses and Input

Next, to couple the neurons via synapses, we add an additional *Behaviour* module, *Input_Behaviour* (see Code block 3). This module collects input at afferent synapses and updates the vector of neurons' voltages accordingly. In *set_variables* the synapse matrix W is created, which stores one weight-value for each connection. *W* has the dimension of *D* × *S*, where *D* is the size of the destination *NeuronGroup* and *S* is the size of the source *NeuronGroup* group. Such synapse matrices can be conveniently created with the function *get_synapse_mat* with equal or random values. The function *new_iteration* defines how the information is propagated through the synapses (dot product). Here, the for-loops are not necessary, because we only have one *SynapseGroup*. However, they would be required for multiple *Neuron*- and *SynapseGroups*. With *synapse.src* and *synapse.dst* you can access the source and destination *NeuronGroups* assigned to a *SynapseGroup*.

In this example, the membrane voltage is mainly driven by random input, which avoids network instability due to runaway excitation. Mechanisms for stabilizing network activity, like a refractory period, intrinsic plasticity, or interneurons can be added with further modules and neuron groups.


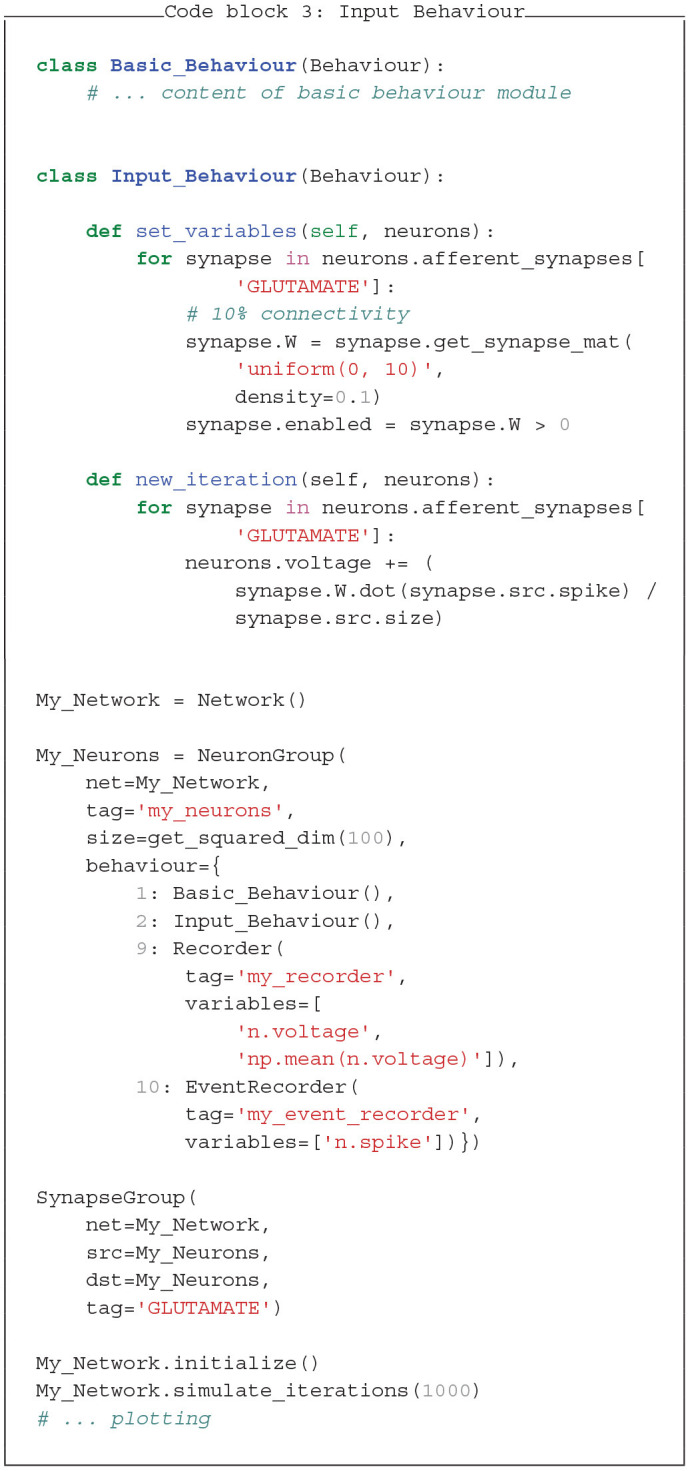


### 3.4. Tagging System and Plotting

PymoNNto's tagging system makes access to the *NeuronGroups, SynapseGroups, Behaviours*, and recorded variables inside the network more convenient. To access the tagged objects we can use the *[]* operator. *['my_tag']* returns a list of all objects tagged with *my_tag*. It basically searches the whole tree structure defined by the object and its children recursively. Because of an internal caching mechanism, the search is only performed once. After the first search, the execution is as fast as a dictionary access when the same tag is requested repeatedly. Therefore it can also be used in *Behaviour* modules where speed is critical.

**Figure 5:**
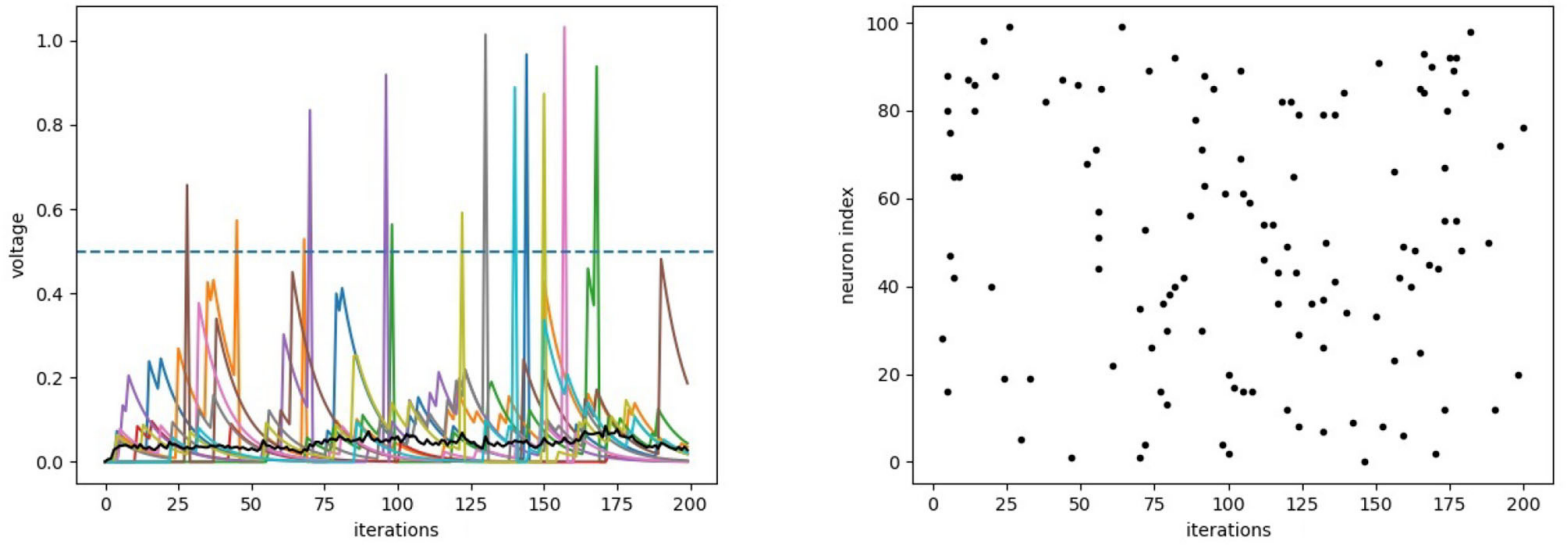
The output of the plotting code block 4 (with additional axis labels), where the neurons receive random input from the *Basic_Behaviour* and additional input from other neurons through the *Input_Behaviour*. **(Left)** The individual voltage traces of the first 10 neurons are plotted with different colors, the mean voltage in black and the (constant) firing threshold with dashed lines. **(Right)** Raster plot showing spikes (black dots) of all neurons during the same period.


In the following Code block 4 we see an example of how the tagging system can be used to plot data. Here we access the variables stored in the recorder from the previous example after the simulation. An example output of this code is shown in Figure 5. Internally, the recording strings *"n.voltage"* and *"np.mean(n.voltage)"* are converted into Python code and executed at every time step during recording. These strings also act as tags for the tagging system to access the recorded data series. In the code block, we also show some general examples and their output to illustrate how the tagging system can be used.

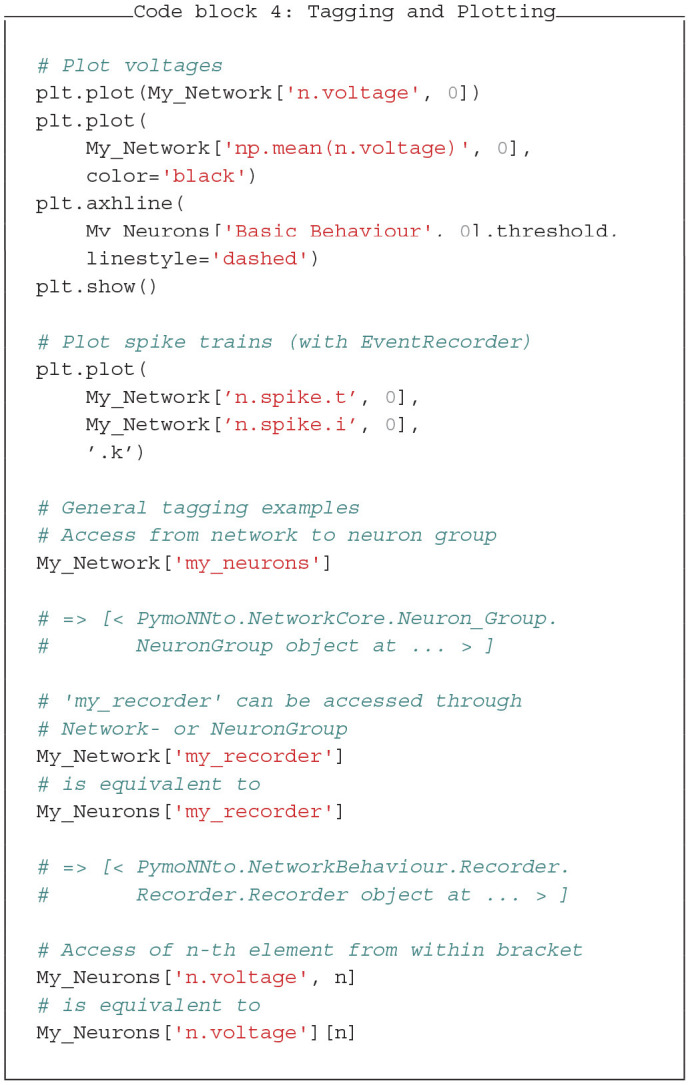


### 3.5. Diversification and Initialization

The *Basic_Behaviour* code example can also be extended with another useful feature of PymoNNto. *Behaviour* modules provide additional functions for compact behaviour initialization. Because it can be useful to access the parent object during the initialization, *Behaviour* modules do not use the typical Python class constructor *init*. The problem with the default constructor is that the parent neuron group is not yet created when the behaviour is constructed. To solve this, variables are initialized in *set_variables*, which is called at the end of the network description. Here, the *get_init_attr* function allows to access the original initialization attributes. Further, the *get_init_attr* function adds functionality for neuron diversification. In the code example *leak_factor* is a number. However, when we, for example, change the initialization to *Basic_Behaviour[leak_factor='normal(0.9,0.1);plot']*, the variable becomes a vector with different values for each neuron, without changing the rest of the code. In this example we use a normal distribution, which can be displayed in a histogram with the optional ";plot" string at the end. We can use all distributions in the numpy.random package, like lognormal, uniform, or poisson, as well as custom functions.

### 3.6. Graphical User Interface

**Figure 6:**
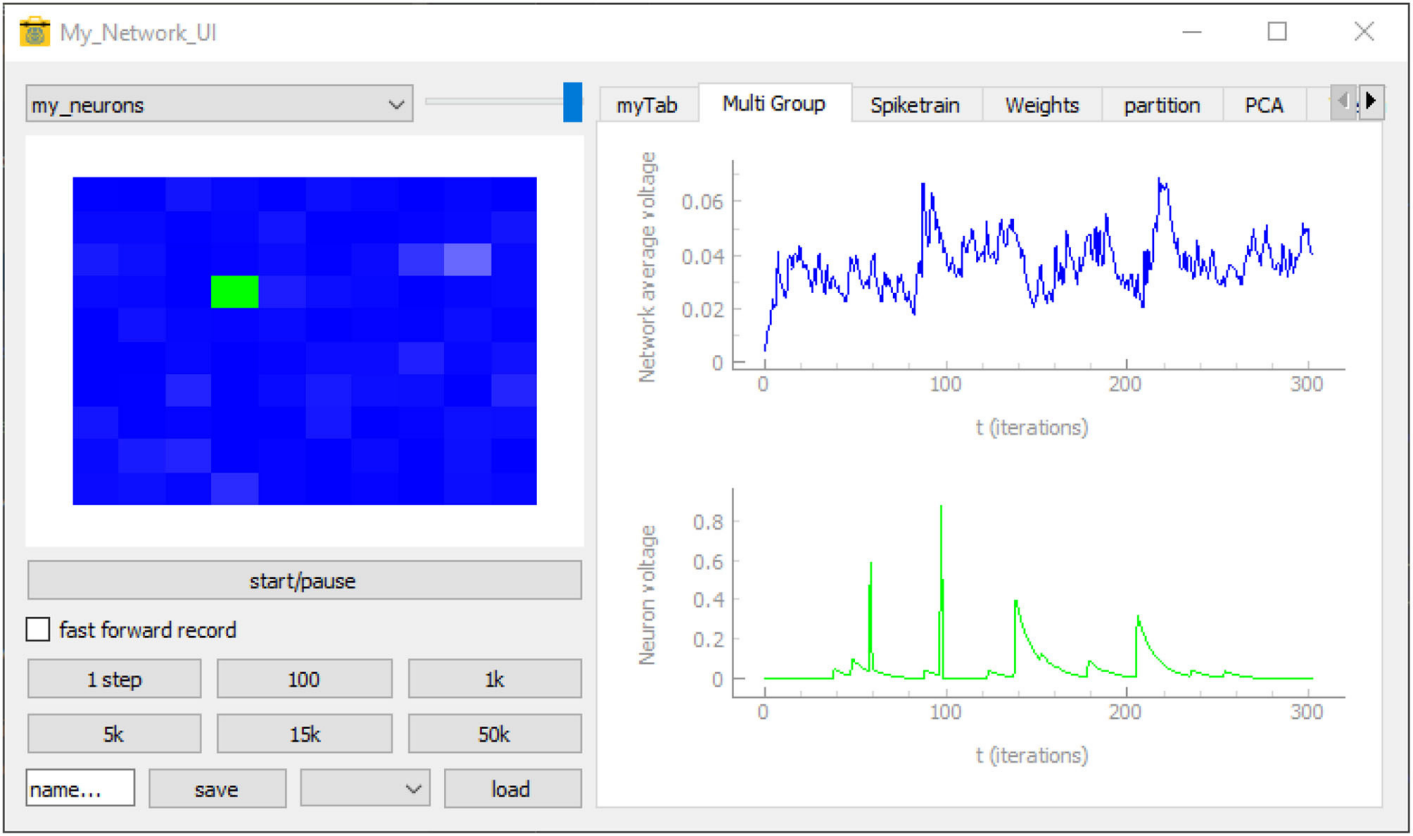
An image of the graphical user interface executed with the code from the User Interface section. The neuron grid on the left displays the activity of each neuron by increasing levels of brightness. The control panel below includes controls to start, pause, save, and load the simulation. The *Multi Group* tab that has been selected on the right displays the mean activity (blue trace) of the whole neuron group as well as the activity (green trace) of a selected neuron (green pixel in neuron grid) across time. The other (non-selected) tabs listed at the top provide additional forms of live visualizations when selected.


To control and evaluate our model with PymoNNto's interactive graphical user interface we can replace the *pyplot* functions (Hunter, 2007), the *recorder* and the *simulate_iterations* with code to launch the *Network_UI* (Code block 5 and Figure 6). Like other parts of PymoNNto, the *Network_UI* is modular. It consists of multiple *UI_modules*, which can be freely chosen. Here, we use the function *get_default_UI_modules* to get a list of standard modules applicable to most networks. To correctly render the output, some *UI_modules* require additional specifications or adjustment of the code. In this example, the *sidebar_activity_module* displays the activity of the neurons on a grid and allows to select individual neurons (blue rectangle, Figure 5). The size is specified via a *NeuronDimension* behaviour, which receives the width, height and depth of the grid and creates spatial coordinates for each neuron stored in the vectors *x*, *y*, and *z*.

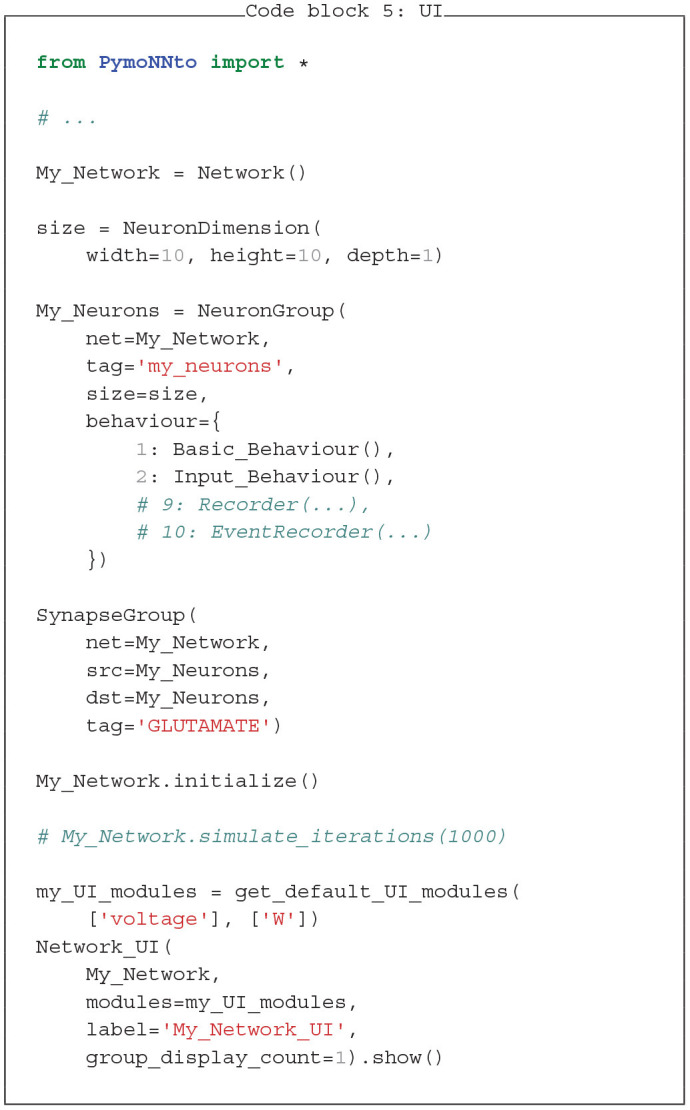


## 4. Flexibility

So far, the presented examples relied on NumPy (Harris et al., 2020) routines for data storage and computation. However, the minimal design of PymoNNto allows to freely define and optimize data types and computations to any specific problem. Any Python-based data representation or computation library can be employed, such as PyTorch matrices or SciPy sparse matrices.

### 4.1. Increase of Simulation Speed With Tensorflow

To demonstrate PymoNNto's versatility, we re-implement the examples of section 3 with Tensorflow 2 (see Code block 6). Commonly used for deep learning, Tensorflow efficiently operates with tensor graphs, which are multidimensional arrays, connected by mathematical operations. These operations are not restricted to deep learning approaches and rather resemble NumPy's functionality, with only few exceptions.

The use of Tensorflow can substantially increase simulation speed for large networks (Mohanta and Assisi, 2019). Tensorflow is highly optimized and natively runs on CPUs, GPUs or even specialized Tensor Processing Units.

**Figure 7:**
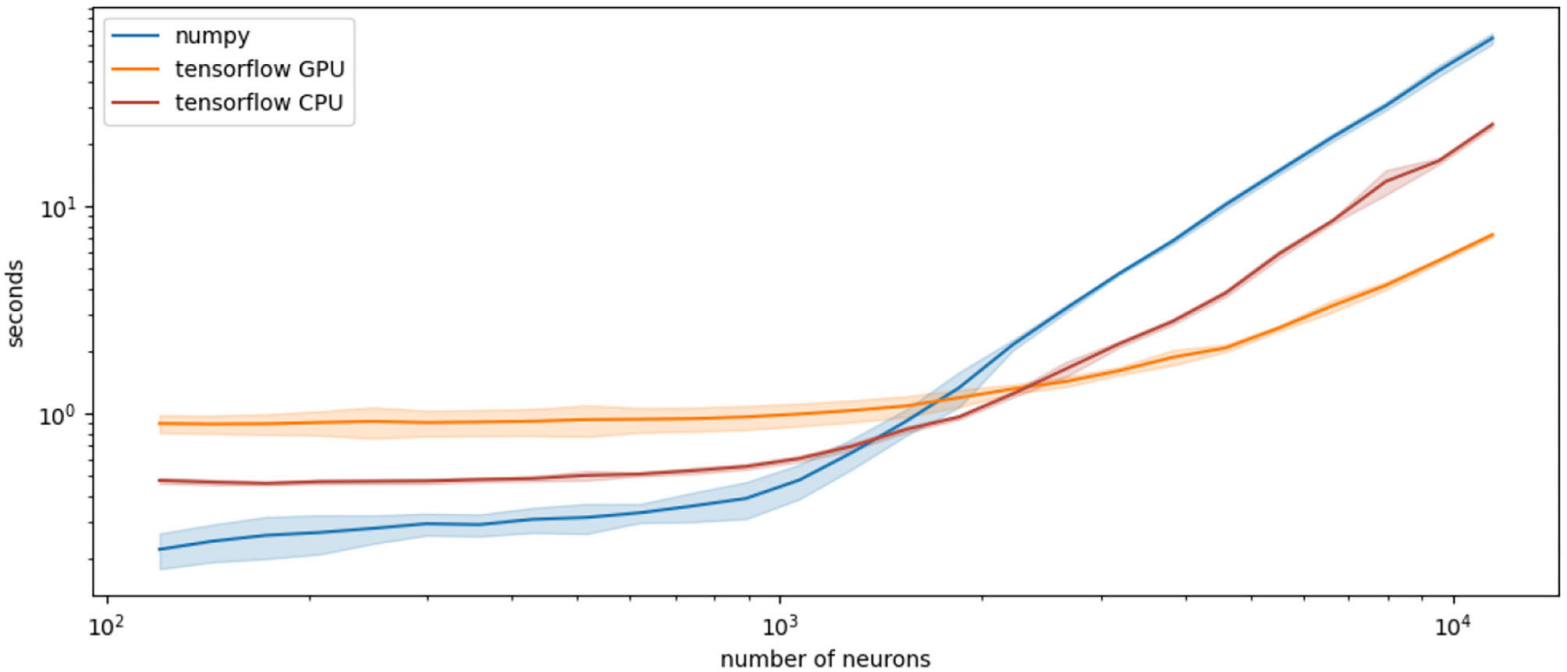
Comparison of processing time (y axis, log scale, seconds) for the described network comprised of different numbers of neurons (x axis, log scale, number of neurons) implemented either with NumPy (blue) or Tensorflow (orange and red) modules. Plotted are the means over 10 runs and their standard deviations.

To compare the performance, we simulated the neural network, defined as NumPy version in section 3, and its Tensorflow counterpart with different sizes (~10^2^−10^4^ neurons in steps of 100 * 1.2s; 1,000 iterations; computed on a Dell XPS 15 with i7-8750H CPU and Nvidia-GeForce-GTX-1050-Ti GPU). We find that Tensorflow is slower compared to NumPy for small networks (below around 2,000 neurons), likely due to its larger computational overhead. However, for larger networks, Tensorflow is consistently faster on both, the CPU and GPU (see Figure 7). Note, the speed of the Tensorflow network may be further optimized. Especially, the creation and conversion of a new random vector at every time step is not optimal, but it makes the comparison to the NumPy implementation easier.

The mixing of NumPy and Tensorflow modules is also possible but requires conversions with the *tensor.numpy()* command. This, however, only makes sense when only small vectors are moved from GPU to CPU memory and back. One potentially useful option would be to shift the computationally expensive weight matrix and its operations to the GPU via Tensorflow, while only the result vectors are moved to the CPU for further processing.


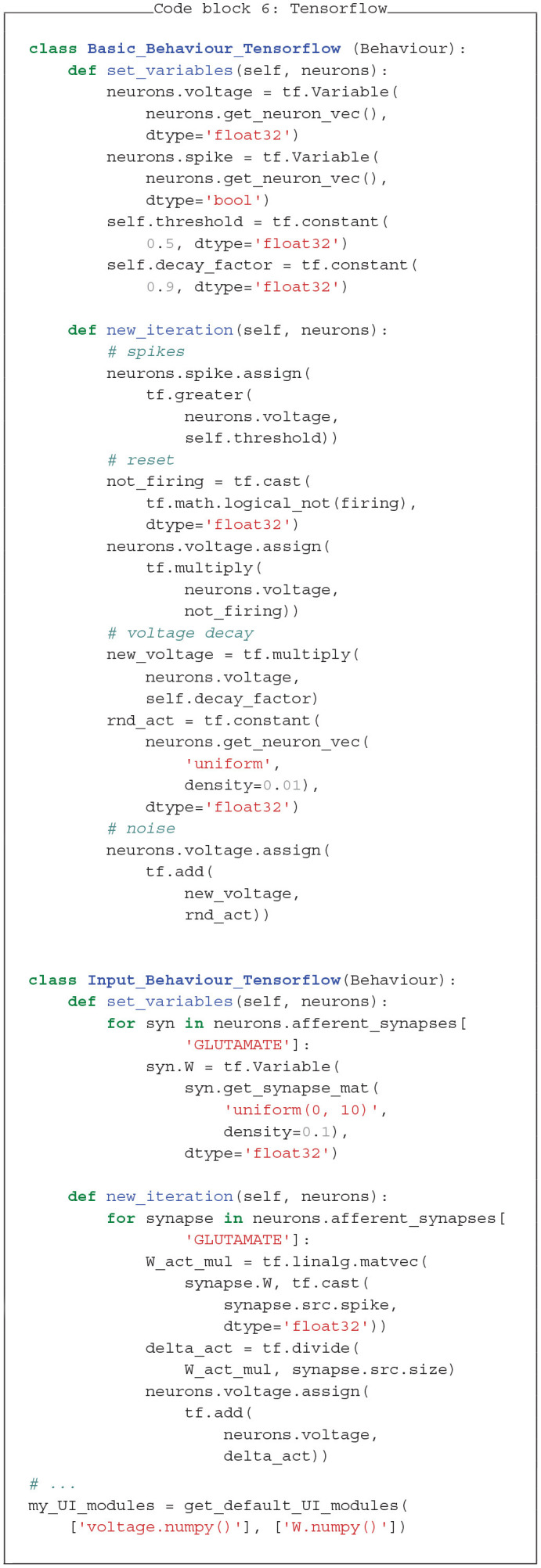


### 4.2. PymoNNto Supports Brian2-Like Model Definition

A major advantage of Brian2 is its concise model definition. Dynamics are defined as a string of differential equations with physical units handled by the SymPy package (Meurer et al., 2017). In Code blocks 7 and 8 we show how similar features can be added to PymoNNto with few additional modules: The *Clock* module keeps track of time across iterations, the *Variable* module initializes the neuron parameters and the *Equation* module handles differential equations in string format. While these modules are still in development, they already allow to write PymoNNto programs which resemble Brian2's concise style and produce similar results with similar processing speed.


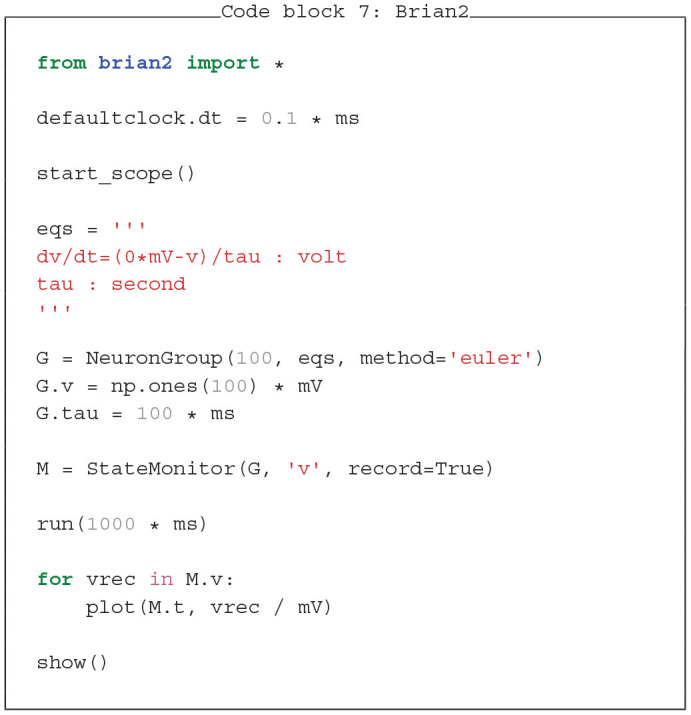



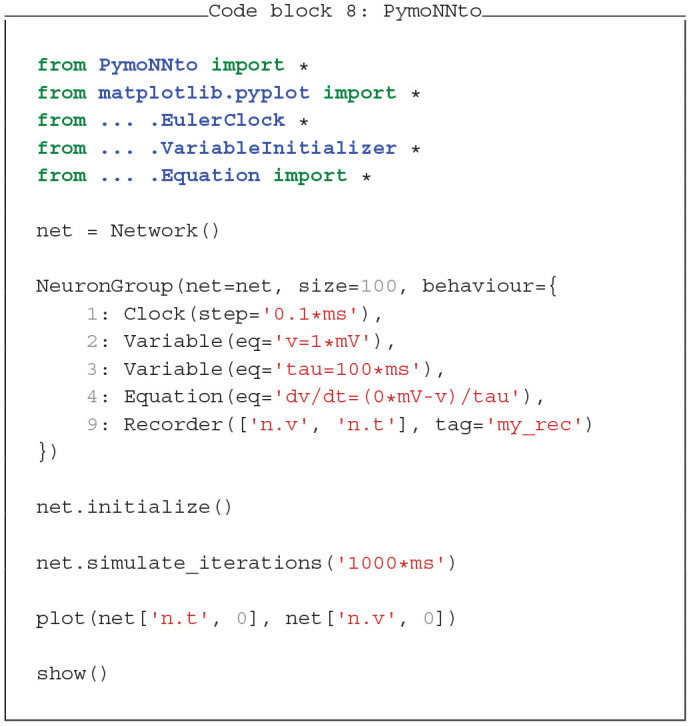


### 4.3. Simulator Fusion With PymoNNto

The flexible and modular nature of PymoNNto allows to embed other simulators into PymoNNto. This unique feature allows to combine the functionality of other simulators with PymoNNto modules and its user interface. For clarity we only show two minimal examples, integrating Brian2 and NEST into PymoNNto (see Code block 9 and 10).


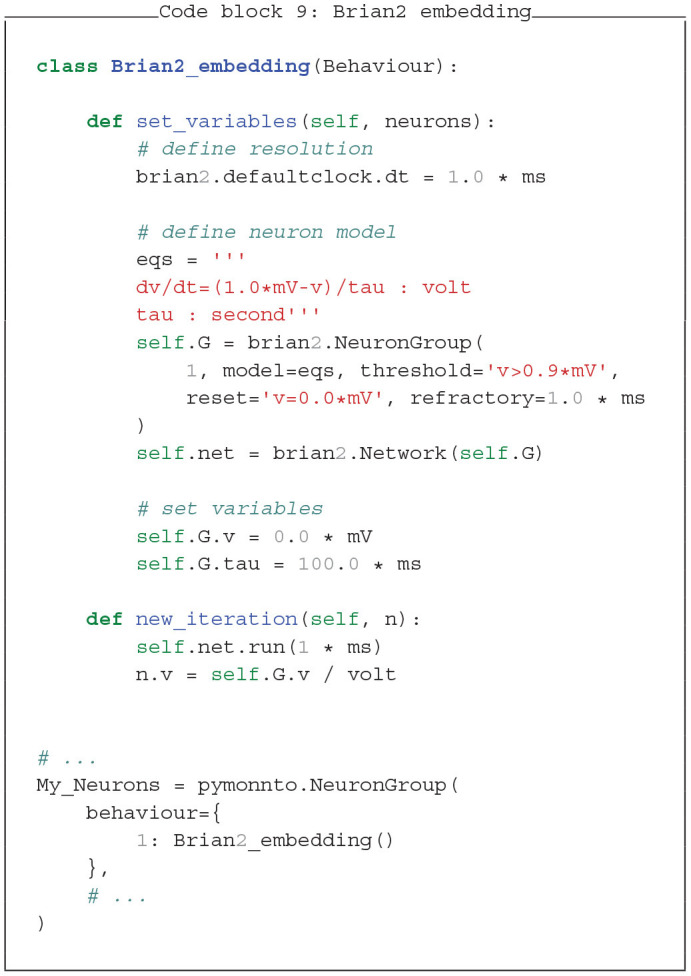



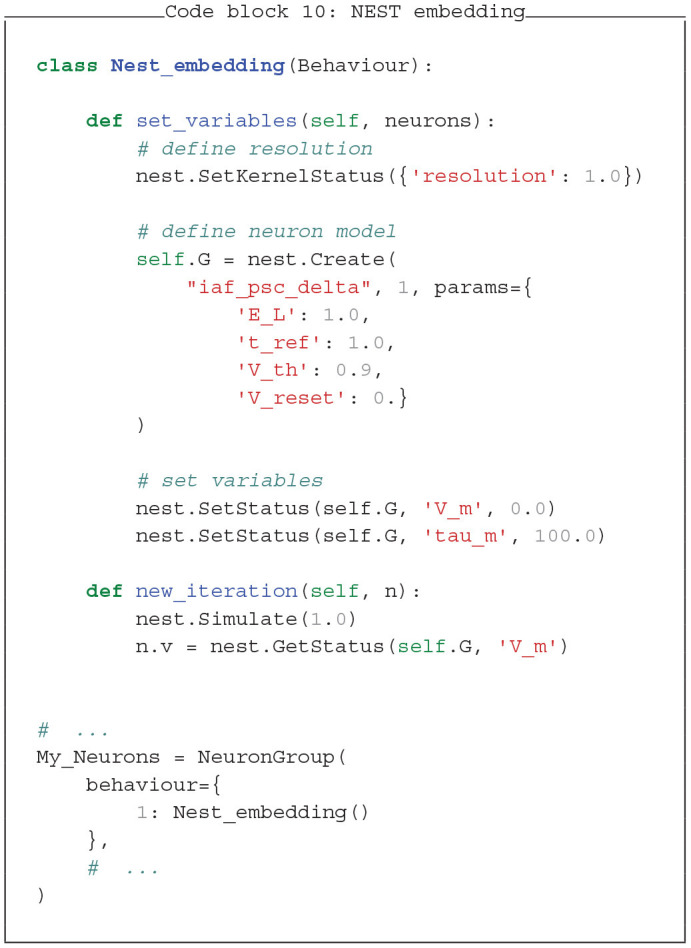


### 4.4. Custom UI Module

In this last example (Code block 11) we define a custom tab for the graphical user interface to plot the mean voltage of the neuron group (compare blue trace in Figure 6). UI modules are derived from the TabBase class, which typically consists of the following four functions: *__init__, add_recording_variables, initialize* and *update*. These modules have a similar layout as *Behaviour* modules. The update function is called at every timestep. To access the parent user interface, we use an additional initialization function. Here, the *__init__* function is only defined to give the tab a name which can be done before the parent user interface is initialized.

We specify the tab and its user interface elements in the *initialize* function. First, we add a new tab by calling *Network_UI.Next_Tab* which creates a new tab element and a corresponding layout for the internal components. This layout is arranged in rows and we can attach Qt widgets (QLabel, QPushButton, QSlider, …) to the current row with the *Add_Element* function. *Next_H_Block* can be called to jump to the next row. In this example we want to add a PyQtGraph plot to the tab, which is also a Qt widget compatible with the rest of the Qt framework. Because plotting is relatively common, there is a convenience function *Add_plot_curve* which creates a plot with a curve and adds them to the current row automatically.

Next, we define the recording variables in the *add_recording_variables* function. To this end, we call the *Network_UI* function *add_recording_variable*, specifying what we want to record and for how many time steps. This function checks whether there are redundant recorders and, if so, replaces them with one recorder covering the full recording time to improve memory efficiency. The access through the tagging system is not affected by this and is still the same as in the previous plotting example. Alternatively, one could directly add a recorder to the neuron group similar to the previous examples. However, this could be inefficient if multiple tabs use partially redundant recorders.

The last step is to define the *update* function which refreshes the plotted voltage trace. To save resources we check whether the tab is visible in the first place. If so, we access the recorded data via the tagging system. Like in the previous plotting example we can use the same string for variable evaluation and tagging. Therefore, *[“np.mean(n.voltage),” 0, “np”]* gives us the recorded mean of the voltage, selects the first and only element in the list of the tagged objects and directly converts it to a numpy array with the “*np”* attribute. The *[*−*1000:]* at the end is optional and ensures that the plotted trace is not longer than 1,000 elements, which could be the case when merging recorders of different length. This, however, only gives us the y-axis data. If we want to get the corresponding time steps on the x-axis, we can access the *n.iteration* trace in the same way as the y-data. This is possible, because the *Network_UI* adds this recorder automatically. To display the custom tab, we can add it to the list of *ui_modules* from the first examples, which is shown at the bottom of the code block.


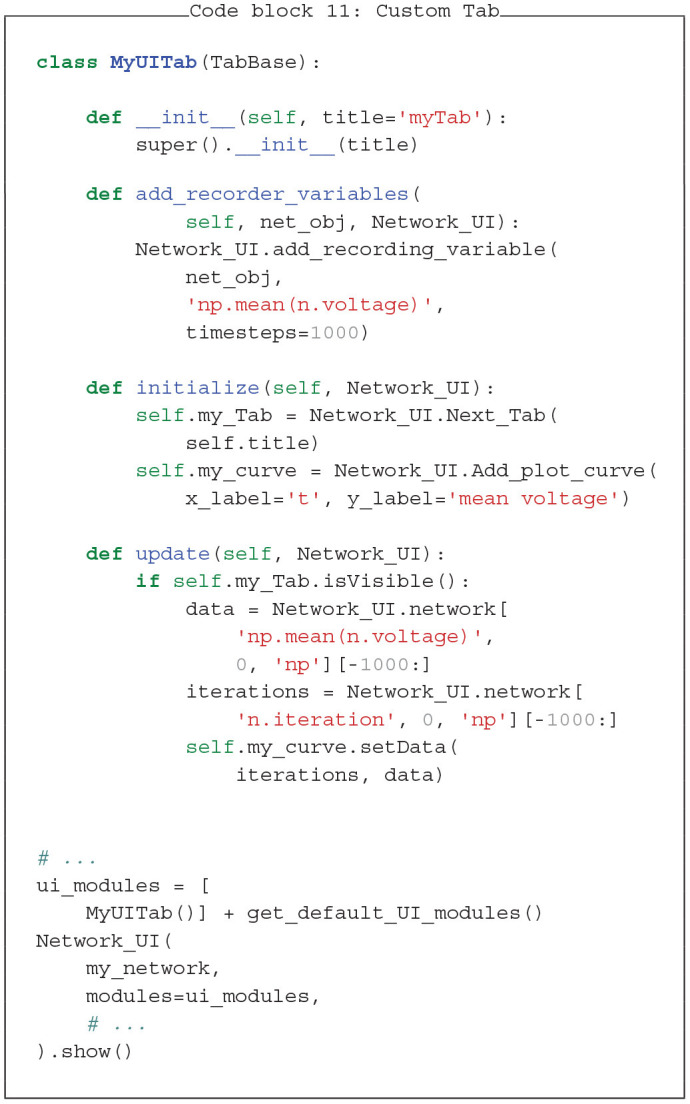


### 4.5. Cython

Performance of Python code can be drastically improved by using Cython, which compiles Python into faster C code. In contrast to the PymoNNto's lightweight core, *Behaviour* modules with their heavy computations may strongly benefit from the use of Cython. The flexibility of PymoNNto allows to speed up only selected *Behaviour* modules. This leaves the rest of the code unaffected and avoids extensive re-compilation at every run. PymoNNto's online documentation contains detailed instructions on how to use Cython with PymoNNto.

## 5. Discussion

We presented PymoNNto, a flexible modular neural network toolbox, which provides a low level core together with several high level features. This design aims to impose only minimal restrictions for model definition, while at the same time simplifying the network development via support functions. The flexibility of PymoNNto allows for any Python-based data representation and computation, opening the way to seamless interactions with external neuronal network libraries, such as Tensorflow or Brian2. Featuring a versatile user interface, a storage manager and an evolution package for hyperparameter tuning, Pymonnto facilitates an efficient workflow.

PymoNNto's emphasis on flexibility defines its niche in the vibrant ecosystem of neural network simulators. In recent years, the research community has been witnessing intensive developments of many established simulators. For example, NEST Desktop allows to design, control and analyse NEST simulations without the need to write code (Spreizer et al., 2021). Going even further, NetPyNE, a simulation manager for Neuron, provides both a programmatic and graphical interface for model definition, standardized import and export, parallel execution, parameter optimization, visualization, and analysis (Dura-Bernal et al., 2019). Making use of the highly-optimized deep learning library PyTorch, BindsNET can efficiently simulate spiking neural networks both on CPUs and GPUs (Hazan et al., 2018). And, the simulator Nengo (Bekolay et al., 2014) recently received a backend for Intel's neuromorphic chip Loihi (Davies et al., 2018). Together, these developments illustrate a common trend: The number of options increases for how to define a model and which hardware to use for execution. However, in most cases, these additions do not extend the expressive power of the respective core. PymoNNto strives not only to allow for flexible core control but also to keep the core itself as flexible as possible.

Due to its simple core design, PymoNNto is easy to learn. Furthermore, transferring existing custom vector based models to PymoNNto is straightforward. Hence, PymoNNto may be of great interest to researchers that, until now, do not use existing simulators for their custom models, because of described restrictions of these simulators. With PymoNNto they get access to a powerful GUI and many useful support and analysis functions with minimal changes to their code.

While we see PymoNNto primarily as a stand-alone neural network simulator, it can also be used in combination with one or several external simulation environments. While in our minimal example PymoNNto was only combined with Brian2 and NEST, more complex interactions can be conceived. For example, a Brian2 NeuronGroup could use a native PymoNNto plasticity module, while it interacts with a deep neural network implemented in Tensorflow. Note, as of now PymoNNto provides only a scaffold for interactions, but does not possess any built-in optimization for such processes. Thus, potential pitfalls remain and users need to assert caution when integrating external libraries. In a related approach, real-time interactions between a robotic simulator and the NEST simulation environment have been achieved by bridging between the Multi-Simulator Coordinator (MUSIC) and the Robotic Operating System (ROS) (Weidel et al., 2016).

When using differential equation-based model definitions, users may choose between PymoNNto's own differential equation module or integrating Brian2 into PymoNNto code. While integrating Brian2 directly allows access to its extensive functionality, PymoNNto's differential equation module has a lower computational overhead and allows for additional flexibility.

The features demonstrated in this manuscript are considered stable except otherwise noted. In the future, we aim to include additional features, such as pre-processing functions for video and audio data, advanced high level modules for convenient model definition, and multicore processing of single networks. Because a single Python instance can execute operations only on a single core, multiprocessing or distributed computing is currently limited to specific cases: Computations can be executed on multiple cores via Tensorflow; and the *Evolution* module uses a “pleasingly parallel” computation scheme to test different parameter configurations on multiple cores and machines. To enable true multiprocessing, we intend to explore data exchange between multiple Python instances or a PymoNNto C++ backend with a Python interface. Another goal is to expand the public repository for behaviour and GUI modules. This would facilitate the incorporation of, e.g., plasticity models or useful network visualization tools developed by other research groups.

PymoNNto facilitates interactions between spiking neural networks and deep learning. The efficiency of training deep non-spiking convolutional networks has led to remarkable progress in artificial intelligence research (LeCun et al., 2015; Schmidhuber, 2015). In contrast, as of now, spiking neural networks are mostly used in the context of brain research. Reflecting this divide, largely different tools are used in each of the two domains. Boosted by the prospect of energy-efficient neuromorphic hardware, efforts have been started to translate deep-learning based training algorithms to spiking neural networks (Pfeiffer and Pfeil, 2018; Zenke and Ganguli, 2018; Neftci et al., 2019, for a review see Tavanaei et al., 2019). In addition, deep learning frameworks are extended to simulate spiking neural networks (Hazan et al., 2018; Mozafari et al., 2019). Thus, with increasing interactions between these two fields, it will become important to have tools like PymoNNto, which can be used in both contexts and can flexibly combine the strengths of existing libraries.

## 6. Development and Availability

PymoNNto is released under the free and open MIT licence (Massachusetts Institute of Technology, 1988). The development is public and code is available at: https://github.com/trieschlab/PymoNNto Tutorials and documentation can be found at: https://pymonnto.readthedocs.io. All code examples can be found in the GitHub repository and were executed with PymoNNto's release version 1 and the library versions from the release description. We invite the community to contribute to PymoNNto's development and to extend the ecosystem with additional behaviour and UI modules.

## Data Availability Statement

Publicly available datasets were analyzed in this study. This data can be found here: https://github.com/trieschlab/PymoNNto.

## Author Contributions

MV is the main software developer of PymoNNto. MV and TS created the figures. MV, TS, and JT wrote the article. JT supervised the project. All authors contributed to the article and approved the submitted version.

## Funding

This work was supported by the European Union, Horizon 2020 Research and Innovation Program, G.A. no. 713010, Project GOAL-Robots—Goal-based Open-ended Autonomous Learning Robots (MV), The German Research Foundation, DFG, SPP 2041, Project number 347573108: The dynamic connectome: keeping the balance (TS) and The dynamic connectome: dynamics of learning (MV), the LOEWE Center for Personalized Translational Epilepsy Research (CePTER) (TS), and the Johanna Quandt foundation (JT).

## Conflict of Interest

The authors declare that the research was conducted in the absence of any commercial or financial relationships that could be construed as a potential conflict of interest.

## Publisher's Note

All claims expressed in this article are solely those of the authors and do not necessarily represent those of their affiliated organizations, or those of the publisher, the editors and the reviewers. Any product that may be evaluated in this article, or claim that may be made by its manufacturer, is not guaranteed or endorsed by the publisher.
